# Initial evaluation of thyroid dysfunction - Are simultaneous TSH and fT4 tests necessary?

**DOI:** 10.1371/journal.pone.0196631

**Published:** 2018-04-30

**Authors:** Claudio Schneider, Martin Feller, Douglas C. Bauer, Tinh-Hai Collet, Bruno R. da Costa, Reto Auer, Robin P. Peeters, Suzanne J. Brown, Alexandra P. Bremner, Peter C. O’Leary, Peter Feddema, Peter J. Leedman, Drahomir Aujesky, John P. Walsh, Nicolas Rodondi

**Affiliations:** 1 Department of General Internal Medicine, Inselspital, Bern University Hospital, University of Bern, Bern, Switzerland; 2 Institute of Primary Health Care (BIHAM), University of Bern, Bern, Switzerland; 3 Departments of Medicine and Epidemiology and Biostatistics, University of California, San Francisco, California, United States; 4 Service of Endocrinology, Diabetes and Metabolism, Lausanne University Hospital, Lausanne, Switzerland; 5 Applied Health Research Centre, Li Ka Shing Knowledge Institute of St Michael's Hospital, Institute of Health Policy, Management and Evaluation, University of Toronto, Toronto, Canada; 6 Department of Internal Medicine, Erasmus Medical Center, Rotterdam, the Netherlands; 7 Department of Endocrinology and Diabetes, Sir Charles Gairdner Hospital, Nedlands, Western Australia, Australia; 8 School of Population Health, The University of Western Australia, Crawley, Western Australia, Australia; 9 School of Women's and Infants' Health, The University of Western Australia, Crawley, Western Australia, Australia; 10 Diagnostica Stago, Doncaster, Victoria, Australia; 11 Medical School, University of Western Australia, Crawley, Western Australia, Australia; 12 UWA Centre for Medical Research, Harry Perkins Institute for Medical Research, Perth, Western Australia, Australia; The University of Tokyo, JAPAN

## Abstract

**Objective:**

Guidelines for thyroid function evaluation recommend testing TSH first, then assessing fT4 only if TSH is out of the reference range (two-step), but many clinicians initially request both TSH and fT4 (one-step). Given limitations of previous studies, we aimed to compare the two-step with the one-step approach in an unselected community-dwelling study population, and develop a prediction score based on clinical parameters that could identify at-risk patients for thyroid dysfunction.

**Design:**

Cross-sectional analysis of the population-based Busselton Health Study.

**Methods:**

We compared the two-step with the one-step approach, focusing on cases that would be missed by the two-step approach, i.e. those with normal TSH, but out-of-range fT4. We used likelihood ratio tests to identify demographic and clinical parameters associated with thyroid dysfunction and developed a clinical prediction score by using a beta-coefficient based scoring method.

**Results:**

Following the two-step approach, 93.0% of all 4471 participants had normal TSH and would not need further testing. The two-step approach would have missed 3.8% of all participants (169 of 4471) with a normal TSH, but a fT4 outside the reference range. In 85% (144 of 169) of these cases, fT4 fell within 2 pmol/l of fT4 reference range limits, consistent with healthy outliers. The clinical prediction score that performed best excluded only 22.5% of participants from TSH testing.

**Conclusion:**

The two-step approach may avoid measuring fT4 in as many as 93% of individuals with a very small risk of missing thyroid dysfunction. Our findings do not support the simultaneous initial measurement of both TSH and fT4.

## Introduction

Thyroid dysfunction is common, particularly in the elderly.[1–3] Elevated levels of thyroid-stimulating hormone (TSH) define hypothyroidism, and decreased TSH levels define hyperthyroidism.[4–7] When TSH levels are out of the reference range, but concentrations of free thyroxine (fT4) fall within the reference range,[8] this is referred to as subclinical thyroid dysfunction. This entity is defined with laboratory testing because thyroid disorders have a wide range of unspecific clinical manifestations.[9]

Thyroid function tests are among the most frequently requested laboratory procedures[10] and are often considered part of a standard work-up in many situations, such as symptoms like fatigue, anemia, confusion or palpitations[11] as well as for regular check-up examination.[12] Since the diagnosis of thyroid dysfunction requires both TSH and fT4 measurements, measuring both at once may seem most efficient (one-step approach). However, TSH is a sensitive marker of thyroid dysfunction, since small changes in fT4 create large changes in TSH.[13] Earlier studies[14, 15] of outpatients in whom pituitary/hypothalamic disease was not expected showed that it was usually unnecessary to measure fT4 when TSH was normal. Most guidelines therefore recommend a two-step approach (the thyroid cascade): TSH should be measured first, then fT4 only if TSH is out of reference range or there is suspicion of abnormal TSH secretion.[12, 14, 16–18]

Despite clear recommendations,[12, 14, 16–18] clinical practice varies greatly, even among experts and national thyroid associations.[19] A United States study of outpatients showed that about half of TSH measurements (47%) were accompanied by an fT4 or total thyroxine (TT4) test.[20] In their 2013 Atlas, the National Health Service of England also published data that showed 46% of TSH measurements were accompanied by an fT4 measurement.[21] Private labs may charge as much as 129 USD to assess fT4,[22] posing an unnecessary burden on patients and health care systems. This burden could drop with the consistent adoption of a two-step approach.[20] We hypothesise that clinicians do not use the two-step approach because they are concerned about missing those with normal TSH but abnormal fT4.

The need for TSH and fT4 testing could be greatly reduced if we were able to identify patients at risk for thyroid dysfunction prior to the first TSH measurement. To identify healthy participants, a clinical prediction score could help. Therefore, we set out to (i) substantiate the claim that the two-step was better than the one-step approach (for the first time in an unselected, community-dwelling study population), and (ii) develop a prediction score based on clinical parameters, to identify patients at risk for thyroid dysfunction, which could provide a novel three-step approach (first prediction score, then TSH, then fT4).

However, all previous studies have been on selected patients and the validity and safety of the two-step approach has never been examined in an unselected, community-dwelling study population.[15, 23, 24] For the first time we compared the one and the two step approaches in an unselected population in a community setting. Furthermore, as there have been no attempts to develop a clinical risk prediction score in a population-based sample, we tried to find a clinical useful prediction score to predict hypo- or hyperthyroidism.

## Materials and methods

### Setting and study population

We conducted a retrospective, cross-sectional analysis of 4843 participants (aged 17 to 89 years) in the 1994 Busselton Health Survey. Busselton is a rural town in Western Australia with a predominantly white, iodine-sufficient population(http://bpmri.org.au). The busselton health survey has been approved by the Human Research Ethics Committee of the Department of Health of Western Australia. All participants gave informed consent to participate in the busslton health surveys. We excluded participants on anti-thyroid medication (5 participants), on levothyroxine (128 participants), whose TSH (7 participants) or fT4 tests were missing (241 participants). A total of 4471 participants were included in the analysis.

### Definition of main exposures, potential confounders and outcomes

Euthyroidism was defined as TSH within the reference range from 0.45 to 4.49 mU/L. Subclinical hypothyroidism was defined as TSH between 4.50 and 19.99 mU/L and fT4 within the reference range. Overt hypothyroidism was defined as TSH ≥ 20 mU/L or TSH between 4.50 and 19.99 mU/L and fT4 below the reference range.[25, 26] Hyperthyroidism was defined as TSH under 0.45 mU/L, and fT4 measurements allowed us to differentiate between overt hyperthyroidism (fT4 above the reference level) and subclinical hyperthyroidism (fT4 within the reference range). All blood samples were collected and frozen in 1994. Measurements were performed on a single platform in three runs (2007, 2011, and 2013), with reagents from a single supplier. The same TSH assay was used consistently throughout.[27] We defined the reference range for TSH (from 0.45 to 4.49 mU/L), based on expert reviews[8, 28] and a 2010 consensus meeting within our consortium (International Thyroid Conference, Paris, France, 2010).[26] Because the fT4 method was re-standardized by the manufacturer between 2007 and 2011, we converted the 2007 measures so they aligned with the 2011 and 2013 measures.[29] For fT4, reference ranges in the Busselton cohort were defined as 11.2 to 20.8 pmol/l.[29]

To determine if the two-step approach was as accurate and as safe as the one-step approach, we first applied the two-step approach, i.e. evaluated TSH only. Those with an abnormal TSH would subsequently have an fT4 assessment, allowing classification of thyroid dysfunction (subclinical vs overt). We then analysed the participants that would have been missed with a two-step approach, i.e. participants with a normal TSH, but an abnormal fT4. We named these conditions either isolated hypo-thyroxinemia or isolated hyper-thyroxinemia and analyzed the distribution of the fT4 values within these groups.

### Assessment of a three-step approach with a clinical prediction score

We developed two clinical risk scores to predict either hypo- or hyperthyroidism (subclinical and overt). All predefined clinical and laboratory parameters of the dataset which could be associated with hypo- or hyperthyroidism were considered potential candidates for inclusion in the analysis. We chose some predictors because previous studies showed clear association between the predictor and thyroid dysfunction (e.g., smoking status,[30] body mass index (BMI),[31] and blood pressure[32]); others were chosen because of their pathophysiological plausibility. We used univariate logistic regression analysis to assess their association with hypo- or hyperthyroidism and judged corresponding p-values significant when they were < 0.05 in likelihood ratio tests. We then conducted a backward stepwise selection (significance p < 0.20) to determine the final variables to include in the prediction model, along with their respective coefficient. With the final multivariable regression models for hypo- or hyperthyroidism we developed two clinical risk scores, by applying a regression beta-coefficient based scoring method.[33] Integer scores were assigned by dividing risk-factor beta-coefficients by the smallest coefficient and rounding up to the nearest integer.

## Results

Of the 4471 participants included in the analysis, a total of 4156 (93%) had a normal TSH, 35 (0.8%) overt hypothyroidism, 86 (1.9%) subclinical hypothyroidism, 170 (3.8%) subclinical hyperthyroidism, and 23 (0.5%) overt hyperthyroidism. One participant’s TSH and fT4 were both below reference range. He was excluded because of potential euthyroid sick syndrome or hypopituitarism. Mean age was 51.1 (age range 16.5–97.3 years); 55.2% were women (Table 1).

**Table 1:** Participant characteristics.

	Overt Hypothyroidism	Subclinical Hypothyroidism	Isolated Hypo-thyroxinemia	Euthyroid State	Isolated Hyper-thyroxinemia	Subclinical Hyperthyroidism	Overt Hyperthyroidism
N = 4471*	35	86	82	3987	87	170	23
Prevalence	0.8%	1.9%	1.8%	89.2%	1.9%	3.8%	0.5%
**Demographics, mean (range)**							
Age[y] mean (range)	59.8 (35.1–85.7)	62.1 (23.9–86.5)	53.6 (19.2–91.4)	48.8 (16.5–97.3)	46.6 (17.8–88.8)	48.3 (18.4–89.9)	47.6 21.2–82.8)
Female sex [n] (%)	9 (25.7%)	29 (33.7%)	38 (46.3%)	1789 (44.9%)	51 (58.6%)	74 (43.5%)	12 (52.2%)
**Biometrics, mean (range)**							
Height [m]	1.63 (1.48–1.88)	1.64 (1.45–1.83)	1.67 (1.45–1.96)	1.68 (1.35–1.99)	1.71 (1.47–1.90)	1.68 (1.46–1.89)	1.67 (1.53–1.89)
Weight [kg]	74.0 (46.8–102.6)	73.4 (44.8–108.2)	72.9 (46.2–130)	72.9 (34.8–142.2)	71.4 (46–108)	72.0 (48.6–107.6)	72.3 (50.2–106.0)
BMI [kg/m2]	26.5 (19.6–36.9)	26.1 (18.9–40.8)	26.4 (18.5–38.4)	25.5 (15.5–45.1)	24.1 (15.9–36.2)	25.5 (19.1–38.8)	26.3 (19.9–33.9)
Systolic BP [mmHg]	123 (96–170)	125 (93–180)	121 (90–182)	122 (77–211)	121 (96–185)	119 (90–215)	123 (91–161)
Diastolic BP [mmHg]	73 (41–97)	74 (57–107)	75 (52–95)	74.00 (21–119)	72.5 (49–98)	73.00 (50–130)	77.50 (59–95)
**Laboratory, mean (range**)							
TSH [mU/L]	11.6 (4.62–129.0)	5.71 (4.52–13.8)	1.73 (0.50–4.34)	1.26 (0.45–4.49)	1.07 (0.45–4.24))	0.35 (0.01–0.44)	0.23 (0.00–0.43)
FT4 [pmol/l]	9.7 (1.6–18.4)	13.5 (11.2–19.2)	10.5 (1.4–11.15)	16.0 (1.4–33.6)	21.8 (20.8–33.6))	16.8 (11.6–20.7)	24.3 (21.2–38.3)
AntiTPO [pmol/l]	239.0 (5–11106)	132.0 (5–6138)	16.6 (5–1000)	13.7 (1.8–10967)	12.0 (5–369)	13.0 (5–1000)	16.00 (5–484)
AntiTPO pos [n](%)	23 (65.7%)	59 (68.6%)	22 (26.8%)	421 (10.6%)	11 (12.6%)	8 (4.7%)	6 (26.1%)
**Habits, n (%)**							
**Smoking Status**							
Past	10 (32.3%)	28 (33.3%)	35 (43.2%)	1124 (29.6%)	27 (33.8%)	50 (31.4%)	7 (33.3%)
Current < 15 cigs/d	0 (0.0%)	1 (1.2%)	3 (3.7%)	225 (5.9%)	9 (11.3%)	19 (11.9%)	0 (0.0%)
Current > = 15 cigs/d	4 (12.9%)	5 (6.0%)	2 (2.5%)	278 (7.3%)	9 (11.3%)	14 (8.8%)	1 (4.8%)
**Alcohol Status**							
Past	3 (8.6%)	11 (13.3%)	7 (8.6%)	359 (9.1%)	6 (7.0%)	12 (7.1%)	2 (8.7%)
Current	31 (88.6%)	61 (73.5%)	69 (85.2%)	3345 (84.9%)	77 (89.5%)	142 (83.5%)	20 (87.0%)
**Medical History**							
Angina, [n] (%)	1 (3.6%)	1 (1.3%)	1 (1.3%)	56 (1.6%)	3 (3.8%)	4 (2.7%)	0 (0.0%)
Myocardial infarction, [n] (%)	1 (3.2%)	1 (1.3%)	4 (5.1%)	74 (2.1%)	1 (1.3%)	5 (3.3%)	0 (0.0%)
Stroke, [n] (%)	1 (3.3%)	4 (5.1%)	4 (5.3%)	91 (2.5%)	1 (1.3%)	4 (2.6%)	2 (9.5%)
Diabetes, [n] (%)	3 (8.6%)	6 (7.0%)	4 (4.9%)	236 (5.9%)	5 (5.7%)	6 (3.5%)	2 (8.7%)

*One patient is not listed because both fT4 and TSH are below reference range.

Abbreviations: Antithyroid Peroxidase Antibody (Anti-TPO), Anti-TPO > 35 pmol/l (Anti-TPO pos), Blood Pressure (BP), Body Mass Index (BMI), Free Thyroxine (fT4), Thyroid-Stimulating Hormone (TSH)

### Accuracy of one and two-step approach

**Fig 1:**
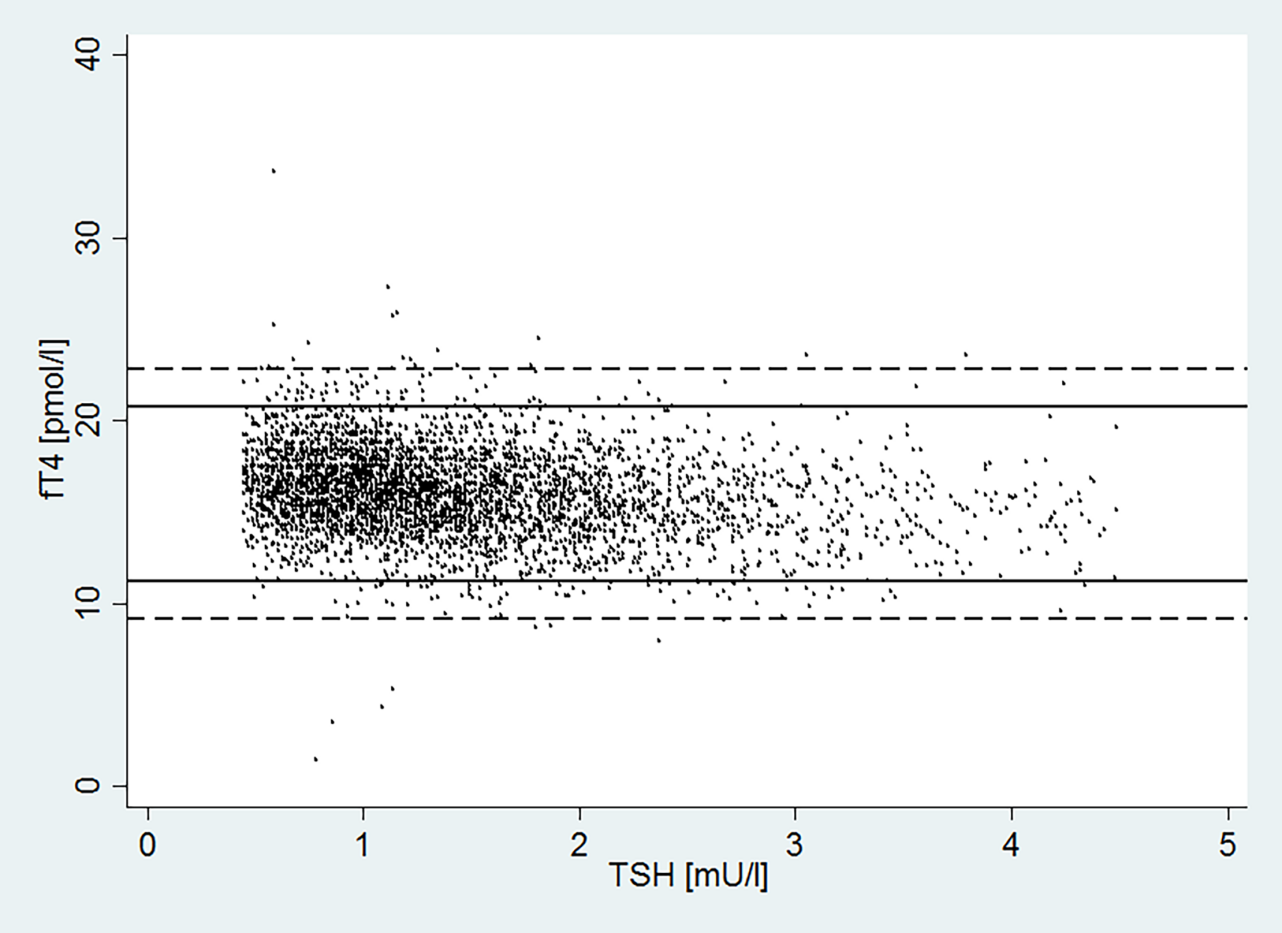
Ft4 measures when TSH falls within the normal range. FT4 measures when TSH falls within the normal range (0.45–4.5 mU/L); 85% of abnormal fT4 measures are within 2 pmol/l (dashed lines) of the reference range (11.2 to 20.8 pmol/L, solid lines). Abbreviations: Free Thyroxine (fT4) Thyroid-Stimulating Hormone (TSH).

Among the 4471 participants, 4156 (93%) had a TSH within the reference range, 3987 (89.2%) had both TSH and fT4 within the reference range. This implies that 96% (3987 / 4156) of the participants with a TSH within the reference are correctly diagnosed as euthyroid by the two-step approach without measuring the fT4. In 169 (3.8%) participants TSH was normal but fT4 was outside the reference range, they comprised 82 participants with isolated hypo-thyroxinemia (normal TSH, decreased fT4) and 87 participants with isolated hyper-thyroxinemia (normal TSH, elevated fT4)(Table 2). Using the two-step approach, these patients would have remained undetected because their TSH was within the reference range. In most, however (85% of the 3.8%) fT4 concentrations were within 2 pmol/l of the reference range limits of fT4 (Fig 1 dashed line). For the remaining 315 (7%) participants with an abnormal TSH, their fT4 would be assessed in any case in the two-step approach.

**Table 2:** Number of individuals with low, normal, and high TSH and FT4.

	TSH, mU/L			
**FT4, pmol/L**	Decreased (<0.45)	Normal (0.45–4.5)	Elevated (>4.5)	Total (%)
Decreased (<11.2)	1	82	33	**116** (2.59%)
Normal (11.2–20.8)	170	3987	88	**4245** (94.95%)
Elevated (>20.8)	23	87	0	**110** (2.48%)
Total (%)	**194** (4.34%)	**4156** (92.95%)	**121** (2.71%)	**4471** (100.00%)

Abbreviations: Free Thyroxine (fT4), Thyroid-Stimulating Hormone (TSH).

### Three-step approach with clinical score development and validation

Univariate likelihood ratio tests to select predictor variables for the multivariate model revealed several that were significantly associated with hypothyroidism (S2 Table). Association with age (p < 0.001) was the most significant. Sex (p = 0.002), height (p = 0.015), and BMI (p = 0.037) were also significantly associated. Predictor variables that were close to statistical significance and included in the prediction model development were systolic blood pressure (p = 0.079), alcohol consumption (p = 0.112), thyroid-affecting medication (p = 0.118, anti-thyroid medication and levothyroxine excluded) and smoking status (p = 0.129). Menopausal state (p < 0.001) was confounded by sex and age, and the association disappeared in multivariate models. Our best model for predicting hypothyroidism only explained about 5 percent of the variation (pseudo R-squared: 0.045). We included the following predictors in the model (with their coefficient in brackets): Age between 50 and 75 years (2 points), older than 75 years (4 points), female (1 point), and BMI ≥ 30 kg/m^2^ (1 point) (S4 Table). The Predictor Score produced a relatively poor area under the curve (AUC) of 0.67 (S1 Fig).

For hyperthyroidism, only smoking status (p = 0.041) was significantly associated with hyperthyroidism (S3 Table), so developing a clinical risk score to detect hyperthyroidism was not feasible.

## Discussion

In this large unselected, community-dwelling population-based study, we showed that the two-step approach (assessing fT4 only if TSH is outside the reference range) could eliminate unnecessary fT4 testing in up to 93% of participants compared with a one-step approach. We could not identify parameters that would allow us to lower the number of TSH measurements by devising a new clinical score that predicts thyroid dysfunction in a clinically useful way.

Clinicians are concerned that the two-step approach may miss some patients with thyroid dysfunction because their TSH value was normal. First, 93.0% of all our study participants had normal TSH; 89.2% had both normal TSH and normal fT4. Thus, 95.9% (89.2% / 93.0%) of those with normal TSH also had normal fT4 levels, and are clearly euthyroid. This proportion matches the three other studies on the topic, which reported proportions of 90.5%,[24] 98.1%,[23] and 99.6%.[15] The high level of agreement across the three studies is reassuring because each included different populations. Ours consisted of unselected, healthy community-dwelling participants, Bauer et al.[23] as well as Viera et al.[15] analyzed patients from an ambulatory care center, and Kende et al.[24] analyzed hospitalized patients. Second, we analyzed patients with normal TSH and fT4 outside the reference range (3.8% of the whole study population) to determine if a sole, normal TSH assessment is likely to miss thyroid dysfunction. We found that most participants (85%) were within 2 pmol/l of the limits of the fT4 reference range. We consider these participants likely to be euthyroid, healthy outliers (the tails of a normal fT4 distribution, S2 Fig). Our interpretation is supported by Bauer et al., who reviewed the charts of those in their hospital-based study population (1.9%) whose TSH value was normal, but fT4 value was abnormal.[23] In no case did measuring fT4 lead to a new diagnosis or change the treatment of a patient. The authors concluded that none of these patients had true thyroid dysfunction.[23] Taken together, ours and previous data show that a sole, normal TSH test reliably diagnoses euthyroidism and does not miss patients with clinically relevant thyroid dysfunction.

Still, despite existing guidelines recommending the two-step approach,[12, 14, 16–18] physicians vary in their approaches to thyroid testing: for example, in Great Britain the rate of test requests varies six-fold between general practitioners.[34] A 2003 study of six French hospitals showed only 62% of indicated thyroid function tests followed published guidelines.[35] Recent research in Wales showed that every third hospital admission gets a thyroid profile.[36] It remains speculative why a significant proportion of physicians still prefers the one-step approach: they may find it more convenient, consider the additional costs of simultaneous fT4 testing negligible, and/or fear to miss relevant thyroid diseases with the two-step approach. There are examples, however, where the two-step approach is well established: In New Zealand, the number of unnecessary thyroid function tests dropped from 56.8% to 34.8% in primary care.[11, 37]^,^ In France, a pre-/post-interventional study that required justification for all laboratory tests increased awareness of thyroid function testing and consecutively decreased simultaneous TSH and FT4 testing from 77% to 51%.[10] Moreover, depending on the local lab habits, biobanking of remaining serum may allow the additional analysis of fT4 from the same blood sample to be ordered only once the abnormal TSH value is known. We assessed TSH in the whole study population (n = 4471) and found that 7% had abnormal TSH values. To reduce the high proportion of unnecessary TSH tests, we tried to develop a score to predict hypo- and hyperthyroidism. The prediction scores we developed could not reliably identify healthy people who don’t need a TSH test. Since both overt and subclinical thyroid dysfunction are associated with adverse health outcomes like coronary heart disease,[26] heart failure,[38] and fractures,[25] we designed our scores to predict combined overt and subclinical thyroid dysfunction. It is possible that the prediction scores we developed did not perform well because combined subclinical and overt thyroid dysfunction reduced prediction. Collecting information about clinical symptoms might have improved our prediction scores, although reported symptoms with subclinical hypothyroidism are not specific.[3, 39] Prior studies yielded conflicting data on this issue.[9, 40–43] Only two studies reported their score’s discriminative ability by measuring the AUC. Canaris et al. reported a poor AUC of 0.64 for their weighted symptom score.[9] Carlé et al’s score effectively identified overt hypothyroidism among young patients (younger men AUC of 0.91, younger women AUC of 0.84), but discriminated poorly for woman above sixty years (AUC 0.64).[41]

The strengths of this analysis lie in the unselected population and the large age range of community-dwelling individuals, which reduces the risk of selection bias and increases the generalisability of our results, at least to white, community-dwelling populations. Furthermore, we added to the body of evidence that shows an initial sole TSH test is sufficient, and fT4 testing is only necessary in a large community-dwelling population if TSH is abnormal. Our study has also limitations. First, our cohort consisted of mostly white, community-dwelling adults, so our results may not apply to all populations. Second, some patients might have been recovering from an acute non-thyroidal illness, leading to the so-called euthyroid sick syndrome or low T3 syndrome.[44] This should affect only a small part of our population, since they were all community-dwelling adults who had to attend a visit at the study centre. Third, the prevalence of subclinical hypothyroidism was somewhat lower, and that of subclinical hyperthyroidism higher than reported previously in this population.[27] This probably reflects the exclusion of individuals on thyroid medication from the current analysis, and the application of a slightly different TSH reference range based on recent publications.[25] Fourth, the two-step approach could have missed central hypothyroidism or hyperthyroidism, or impaired sensitivity to thyroid hormone.[45] In addition to these rare conditions, frequent situations of discordant thyroid function tests include non-thyroidal illness, assay and drug interference.[46] However, most of these clinical situations are accompanied by key clinical symptoms and medical history;[47–49] in such patients, the threshold for fT4 measurement should be low. Fifth, we had no information about clinical symptoms in our study population. We defined subclinical hypothyroidism as TSH between 4.50 and 19.99 mU/L and fT4 within reference range. This is a widely accepted definition of subclinical hypothyroidism and has been used in multiple previous studies.[26, 38, 50, 51] Some experts regard individuals with TSH above 10 mU/L as having overt hypothyroidism,[52] since this is usually an indication for thyroid replacement therapy. We performed a sensitivity analysis that showed similar results with both definitions of overt hypothyroidism (results not shown).

## Conclusion

The guideline-recommended two-step approach assessing fT4 only in case of abnormal TSH may prevent measuring fT4 in as many as 93% of individuals with a very small risk of missing thyroid dysfunction. Our findings do not support the simultaneous initial measurement of both TSH and fT4.

## Supporting information

S1 Fig Sensitivity and specificity of different prediction score levels for classifying hypothyroidism. (TIF)

S2 Fig Density of fT4 values among euthyroids. The red lines indicate reference range limits of fT4 tests measured in this cohort (normal range 11.2–20.8 pmol/l). This graph shows fT4 among euthyroids is normally distributed, and the 169 participants whose TSH is normal but fT4 falls the reference range are predominantly healthy outliers.
(TIF)

S1 Table Prevalence of overt dysfunction within eu-, hypo-, and hyperthyroid groups. (PDF)

S2 Table Univariate analysis of potential predictors of hypothyroidism. (PDF)

S3 Table Univariate analysis of potential predictors of hyperthyroidism. (PDF)

S4 Table Sensitivity and specificity at each score level. (PDF)
