# Supplementary figures and images for: Initial evaluation of thyroid dysfunction - Are simultaneous TSH and fT4 tests necessary?
Source: PLoS One. 2018 Apr 30;13(4):e0196631. doi: 10.1371/journal.pone.0196631 (PMC5927436; doi:10.1371/journal.pone.0196631)

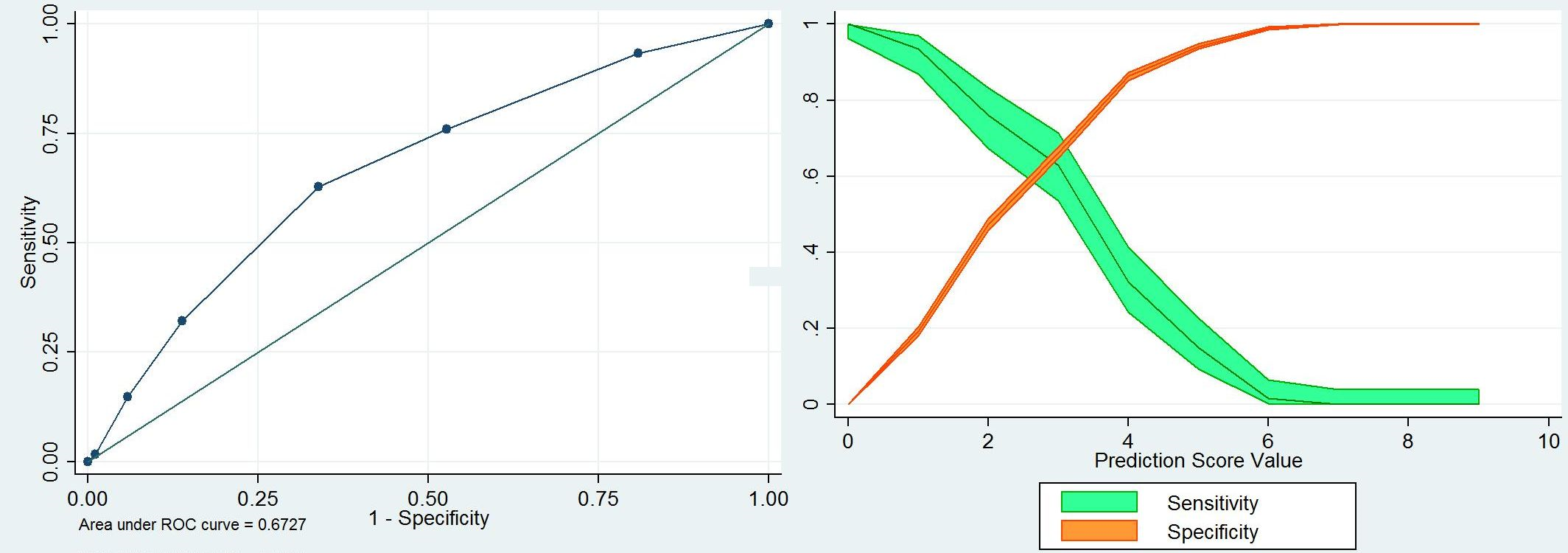

Supplement: S1 Fig — (TIF) [file pone.0196631.s001.tif]

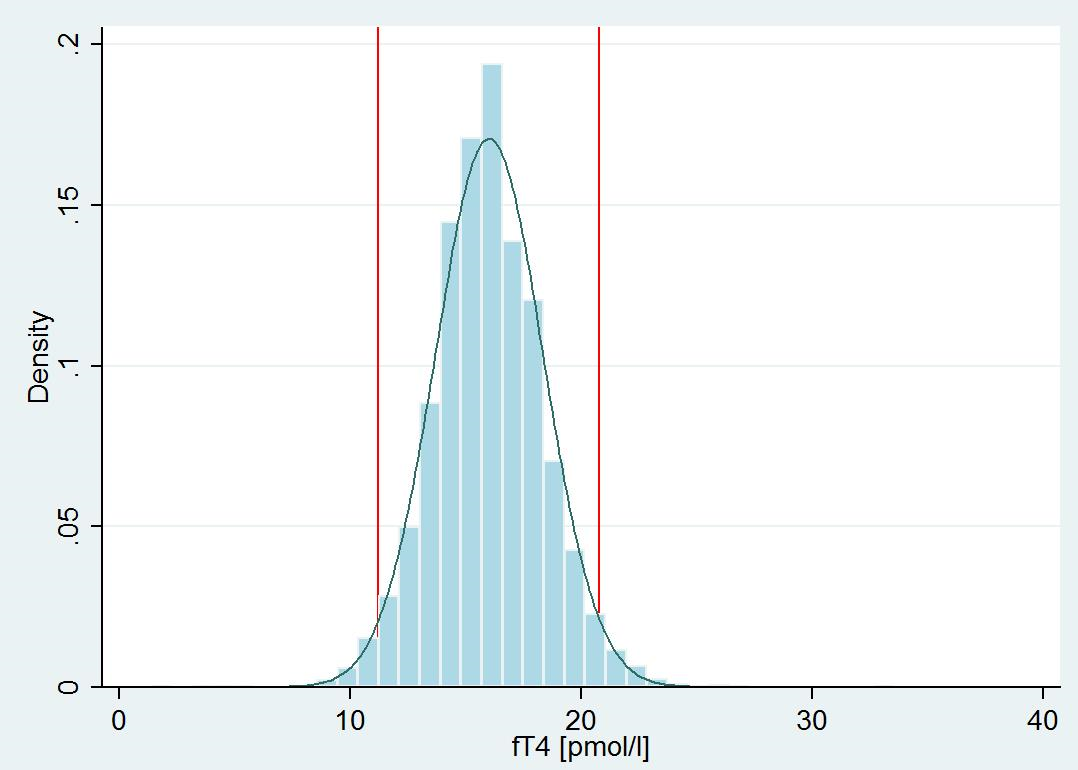

Supplement: S2 Fig — The red lines indicate reference range limits of fT4 tests measured in this cohort (normal range 11.2–20.8 pmol/l). This graph shows fT4 among euthyroids is normally distributed, and the 169 participants whose TSH is normal but fT4 falls the reference range are predominantly healthy outliers. (TIF) [file pone.0196631.s002.tif]
