# Supplementary material for: Initial evaluation of thyroid dysfunction - Are simultaneous TSH and fT4 tests necessary?
Source: PLoS One. 2018 Apr 30;13(4):e0196631. doi: 10.1371/journal.pone.0196631 (PMC5927436; doi:10.1371/journal.pone.0196631)
Supplement: S1 Table — (PDF) [file pone.0196631.s003.pdf]

S1 Table. Prevalence of Overt Dysfunction within eu-, hypo-, and hyperthyroid groups

	**TSH, mU/L**		
	< 0.45	0.45-4.5	>4.5
N = 4471	193	4156	121
Overt Dysfunction	**11.9%(**23)	-	**27.3%** (33)

Abbreviations: Thyroid-Stimulating Hormone (TSH)
