# Supplementary material for: Initial evaluation of thyroid dysfunction - Are simultaneous TSH and fT4 tests necessary?
Source: PLoS One. 2018 Apr 30;13(4):e0196631. doi: 10.1371/journal.pone.0196631 (PMC5927436; doi:10.1371/journal.pone.0196631)
Supplement: S2 Table — (PDF) [file pone.0196631.s004.pdf]

S2 Table. Univariate analysis of potential predictors of hypothyroidism.

Characteristic	Entire cohort	Univariate analysis<br>Hypothyroid State	Euthyroid State	other	P Value
Age					
<50	2286	1.4% (31)	94.1% (2152)	4.5% (103)	< 0.001
50-75	1767	3.8% (67)	92.0% (1626)	4.2% (74)	
>75	418	5.5% (23)	90.4% (378)	4.1% (17)	
Sex					
female	2468	1.9% (38)	93.8% (1878)	4.3% (87)	0.0023
male	2003	3.4% (83)	92.3% (2278)	4.3% (107)	
Weight					
1st quantile	2157	2.7% (58)	92.8% (2002)	4.5% (97)	0.7961
2nd quantile	2192	2.8% (62)	93.1% (2040)	4.1% (90)	
Missing	122	0.8% (1)	93.4% (114)	5.7% (7)	
Height					
1st quantile	2273	3.4% (78)	92.4% (2100)	4.2% (95)	0.015
2nd quantile	2080	2.0% (42)	93.6% (1946)	4.4% (92)	
missing	118	0.8% (1)	93.2% (110)	5.9% (7)	
BMI					
<20	3429	2.5% (84)	93.1% (3194)	4.4% (151)	0.0369
20-30	201	3.0% (6)	94.0% (189)	3.0% (6)	
>30	717	4.2% (30)	91.6% (657)	4.2% (30)	
missing	124	0.8% (1)	93.5% (116)	5.7% (7)	
Smoking					
Never, Past	3681	2.9% (105)	93.2% (3429)	4.0% (147)	0.1288
Current	570	1.8% (10)	92.3% (526)	6.0% (34)	
missing	220	2.7% (6)	91.4% (201)	5.9% (13)	
Alcohol					
Never	274	4.4% (12)	89.4% (245)	6.2% (17)	0.1121
Past	400	3.5% (14)	93.0% (372)	3.5% (14)	
Current	3746	2.4% (92)	93.2% (3491)	4.4% (163)	
missing	51	5.9% (3)	94.1% (48)	0.0% (0)	
Thyroid affecting medication					
No medication	4433	2.6% (118)	93.0% (4121)	4.4% (194)	0.1182
medication	38	7.9% (3)	92.1% (35)	0.0% (0)	
Menopause					
Premenopausal	1217	1.6% (19)	93.8% (1142)	4.6% (56)	< 0.0001
Postmenopausal	1251	5.1% (64)	90.8% (1136)	4.1% (51)	
missing or male	2003	1.9% (38)	93.8% (1878)	4.3% (87)	
Systolic Bloodpressure					
< 140	3587	2.5% (91)	93.0% (3335)	4.5% (161)	0.0785
>= 140	772	3.8% (29)	92.7% (716)	3.5% (27)	
missing	112	0.9% (1)	93.8% (105)	5.3% (6)	
Diastolic Bloodpressure					
< 80	3089	2.8% (88)	92.6% (2860)	4.6% (141)	0.5157
>= 80	1269	2.5% (32)	93.8% (1190)	3.7% (47)	
missing	113	0.9% (1)	93.8% (106)	5.3% (6)	
Diabetes Mellitus					
Negative	4209	2.7% (112)	92.9% (3911)	4.4% (186)	0.4944
Positive	262	3.4% (9)	93.5% (245)	3.1% (8)	

Abbreviations: Body Mass Index (BMI)
