# Supplementary material for: Initial evaluation of thyroid dysfunction - Are simultaneous TSH and fT4 tests necessary?
Source: PLoS One. 2018 Apr 30;13(4):e0196631. doi: 10.1371/journal.pone.0196631 (PMC5927436; doi:10.1371/journal.pone.0196631)
Supplement: S3 Table — (PDF) [file pone.0196631.s005.pdf]

S3 Table. Univariate analysis of potential predictors of hyperthyroidism.

Characteristic	Entire cohort	Univariate analysis: Hyperthyroid State	Euthyroid State	other	P Value
Age					
<50	2286	4.5% (103)	94.1% (2152)	1.4% (31)	**0.6958**
50-75	1767	4.1% (73)	92.0% (1626)	3.9% (68)	
>75	418	4.1% (17)	90.4% (378)	5.5% (23)	
Sex					
female	2003	4.3% (86)	93.8% (1878)	1.9% (39)	**0.8639**
male	2468	4.3% (107)	92.3% (2278)	3.4% (83)	
Weight					
1st quantile	2157	4.5% (96)	92.8% (2002)	2.7% (59)	**0.5785**
2nd quantile	2192	4.1% (90)	93.1% (2040)	2.8% (62)	
missing	122	5.7% (7)	93.5% (114)	0.8% (1)	
Height					
1st quantile	2273	4.2% (95)	92.4% (2100)	3.4% (78)	**0.8252**
2nd quantile	2080	4.4% (91)	93.6% (1946)	2.0% (43)	
missing	118	5.9% (7)	93.2% (110)	0.8% (1)	
BMI					
<20	3429	4.4% (150)	93.1% (3194)	2.5% (85)	**0.7577**
20-30	201	3.0% (6)	94.0% (189)	3.0% (6)	
>30	717	4.2% (30)	91.6% (657)	4.2% (30)	
missing	124	5.7% (7)	93.5% (116)	0.8% (1)	
Smoking					
Never, Past	3681	4.0% (146)	93.2% (3429)	2.9% (106)	**0.0407**
Current	570	6.0% (34)	92.3% (526)	1.7% (10)	
missing	220	5.9% (13)	91.4% (201)	2.7% (6)	
Alcohol					
Never	274	6.2% (17)	89.4% (245)	4.4% (12)	**0.3383**
Past	400	3.5% (14)	93.0% (372)	3.5% (14)	
Current	3746	4.3% (162)	93.2% (3491)	2.5% (93)	
missing	51	0.0% (0)	94.1% (48)	5.9% (3)	
Thyroid affecting medication					
No medication	4433	4.3% (193)	93.0% (4121)	2.7% (119)	**NA**
medication	38	0.0% (0)	92.1% (35)	7.9% (3)	
Menopause					
Premenopausal	1217	4.6% (56)	93.8% (1142)	1.6% (19)	**0.6557**
Postmenopausal	1251	4.1% (51)	90.8% (1136)	5.1% (64)	
missing or male	2003	4.3% (86)	93.8% (1878)	1.9% (39)	
Systolic blood pressure					
< 140	3587	4.5% (160)	93.0% (3335)	2.5% (92)	**0.2439**
>= 140	772	3.5% (27)	92.7% (716)	3.8% (29)	
missing	112	5.4% (6)	93.7% (105)	0.9% (1)	
Diastolic blood pressure					
< 80	3089	4.5% (140)	92.6% (2860)	2.9% (89)	**0.2051**
>= 80	1269	3.7% (47)	93.8% (1190)	2.5% (32)	
missing	113	5.3% (6)	93.8% (106)	0.9% (1)	
Diabetes mellitus					
Negative	4209	4.4% (185)	92.9% (3911)	2.7% (113)	**0.2867**
Positive	262	3.1% (8)	93.5% (245)	3.4% (9)	

Abbreviations: Body Mass Index (BMI), Not applicable (NA)
