# Supplementary material for: Initial evaluation of thyroid dysfunction - Are simultaneous TSH and fT4 tests necessary?
Source: PLoS One. 2018 Apr 30;13(4):e0196631. doi: 10.1371/journal.pone.0196631 (PMC5927436; doi:10.1371/journal.pone.0196631)
Supplement: S4 Table — (PDF) [file pone.0196631.s006.pdf]

S4 Table. Sensitivity and specificity at each score level.

Score Level	Sensitivity		Specificity	
	Fraction (%)	95% CI	Fraction (%)	95% CI
> 0	121/121 (100%)	121/121 (100%)	0/4156 (0%)	0.0-0.1%
> 1	113/121 (93.4%)	113/121 (93.4%)	796/4156 (19.2%)	18.0-20.4%
> 2	92/121 (76.0%)	92/121 (76.0%)	1966/4156 (47.3%)	45.8-48.8%
> 3	76/121 (62.8%)	76/121 (62.8%)	2750/4156 (66.2%)	64.7-67.6%
> 4	39/121 (32.2%)	39/121 (32.2%)	3584/4156 (86.2%)	85.1-87.3%
> 5	18/121 (14.9%)	18/121 (14.9%)	3913/4156 (94.2%)	93.4-94.8%
> 6	2/121 (1.7%)	2/121 (1.7%)	4112/4156 (98.9%)	98.6-99.2%
> 7	0/121 (0%)	0/121 (0%)	4156/4156 (100%)	99.9-100%

Prediction Score	
Characteristic	Points
Age 50-75 y	2
Age ≥ 75 y	4
Women sex	1
BMI ≥ 30 kg/m^2	1

Abbreviations: Body Mass Index(BMI), Confidence Interval (CI), Years (y)
